# ECM proteins in a microporous scaffold influence hepatocyte morphology, function, and gene expression

**DOI:** 10.1038/srep37427

**Published:** 2016-11-29

**Authors:** Yan Wang, Myung Hee Kim, Hitomi Shirahama, Jae Ho Lee, Soon Seng Ng, Jeffrey S. Glenn, Nam-Joon Cho

**Affiliations:** 1 School of Materials Science and Engineering, Nanyang Technological University, 50 Nanyang Avenue, 639798, Singapore; 2 Centre for Biomimetic Sensor Science, Nanyang Technological University, 50 Nanyang Drive, 637553, Singapore; 3 Division of Gastroenterology and Hepatology, Department of Medicine, Stanford University School of Medicine, Palo Alto, California, United States of America; 4 Department of Microbiology and Immunology, Stanford University School of Medicine, Palo Alto, California, United States of America; 5 School of Chemical and Biomedical Engineering, Nanyang Technological University, 62 Nanyang Avenue, 637459, Singapore

## Abstract

It is well known that a three-dimensional (3D) culture environment and the presence of extracellular matrix (ECM) proteins facilitate hepatocyte viability and maintenance of the liver-specific phenotype *in vitro*. However, it is not clear whether specific ECM components such as collagen or fibronectin differentially regulate such processes, especially in 3D scaffolds. In this study, a series of ECM-functionalized inverted colloidal crystal (ICC) microporous scaffolds were fabricated and their influence on Huh-7.5 cell proliferation, morphology, hepatic-specific functions, and patterns of gene expression were compared. Both collagen and fibronectin promoted albumin production and liver-specific gene expression of Huh-7.5 cells, compared with the bare ICC scaffold. Interestingly, cells in the fibronectin-functionalized scaffold exhibited different aggregation patterns to those in the collagen-functionalized scaffold, a variation that could be related to the distinct mRNA expression levels of cell adhesion-related genes. Based on these results, we can conclude that different ECM proteins, such as fibronectin and collagen, indeed play distinct roles in the phenotypic regulation of cells cultured in a 3D environment.

Three-dimensional (3D) cell culture platforms have grown in popularity and recognition in recent decades, especially in the fields of tissue engineering, disease modeling, and drug screening^1^,^2^,^3^,^4^. It is widely accepted that a well-designed 3D culture containing abundant cell-cell and cell-ECM (extracellular matrix) interactions can recapitulate complex *in vivo* physiological constructions and tissue microenvironments, thereby serving as a better *in vitro* model than a conventional 2D cell culture^1^. For example, hepatocytes rapidly lose their specific function and phenotype when they are cultured on 2D plates^5^,^6^ but they can maintain their function and polarity in 3D culture platforms due to the presence of various mechanical and environmental cues^2^,^7^. Yarmush and colleagues developed the collagen sandwich format for the culture of rat hepatocytes^7^,^8^,^9^,^10^. They cultured the cells between two layers of collagen, the major ECM protein, and were able to reconstruct the hepatocyte polarity found in *in vivo* physiology and to maintain hepatic function for as long as 6 weeks. Kotov and his team reported the controlled formation of uniformly-sized liver tumor cell spheroids in inverted colloidal crystal (ICC) scaffolds with uniform porosity^11^,^12^,^13^,^14^,^15^, as well as cells displaying morphological similarities to liver tissue such as bile canaliculi^14^.

Huh-7.5 is a hepatocellular carcinoma cell line that is highly permissive to hepatitis C virus (HCV) infection, and it has been widely used as a model liver cell for tissue engineering and HCV infection studies^16^,^17^,^18^,^19^,^20^,^21^. In our previous study, we fabricated a biofunctionalized hydrogel microscaffold for the culture of Huh-7.5 cells^22^,^23^. The formation of a multilayer hepatic sheet on the surface of collagen-functionalized microscaffold cavities was observed, in contrast to a spheroid formation upon plating into a non-coated scaffold. In addition to a different morphology, conjugated collagen also elicited favorable hepatic functions, such as albumin production, and regulated a different gene expression pattern compared to culture on either a 2D monolayer or on a bare scaffold as spheroids. The important role of ECM proteins in hepatocyte survival and function has also been demonstrated by a number of other reports^24^,^25^,^26^.

Multiple important questions arise from these studies, such as whether collagen functionalization is unique and critical for the phenotypic regulation of cells in the microscaffold and whether other ECM proteins have similar and/or distinct effects. The hepatic extracellular matrix is mainly composed of not only collagens (type I, III, IV, V, and VI) but also other proteins including fibronectin, laminin, and proteoglycans^27^. Our results^22^,^23^ along with studies conducted by other groups^8^,^28^,^29^,^30^,^31^ demonstrated that collagen may be important for cell viability and maintenance of hepatocyte function due to enhanced attachment and mechanical regulation. Rubin *et al*. demonstrated that rat hepatocytes attached equally well to all collagen types (type I, II, III, IV and V)^32^, so collagen I is not unique in its role of promoting cell attachment and function. Furthermore, many articles have pointed out that fibronectin plays a different role to collagen in hepatocyte-substrate adhesion and hepatic function^33^,^34^,^35^,^36^, although contradictory evidence also exists^37^, and the exact underlying mechanisms are still elusive. For example, it was reported that fibronectin enhanced the spreading of rat hepatocytes in culture^33^,^34^ and that cells cultured on a fibronectin substrate displayed much more active DNA synthesis than cells cultured on other ECM proteins, including collagen^35^. Hepatocytes cultured on fibronectin presented higher levels of liver-specific gene expression such as albumin and alpha-fetoprotein^36^. Mooney *et al*., however, suggested that hepatocytes maintained high levels of albumin gene expression and liver-specific protein excretion regardless of the type of ECM molecule used for cell attachment^37^.

The goal of the current study was to elucidate whether fibronectin might play a different role to collagen in the regulation of the hepatocyte phenotype in a 3D microporous scaffold. We fabricated collagen I- and fibronectin-functionalized ICC scaffolds to examine the loading efficiency and proliferation of Huh-7.5 hepatocellular carcinoma cells in the microporous scaffolds. Liver-specific functions and cell adhesion patterns were compared at both the protein and gene expression levels. As a result, we identified a remarkably different pattern of growth, adhesion, and function of Huh-7.5 cells on the fibronectin-functionalized scaffold, providing clear evidence that different ECM proteins have distinctive roles in the phenotypic regulation of liver cells in a 3D microporous scaffold.

## Results

### Fabrication and characterization of ECM-functionalized ICC scaffolds

**Figure 1:**
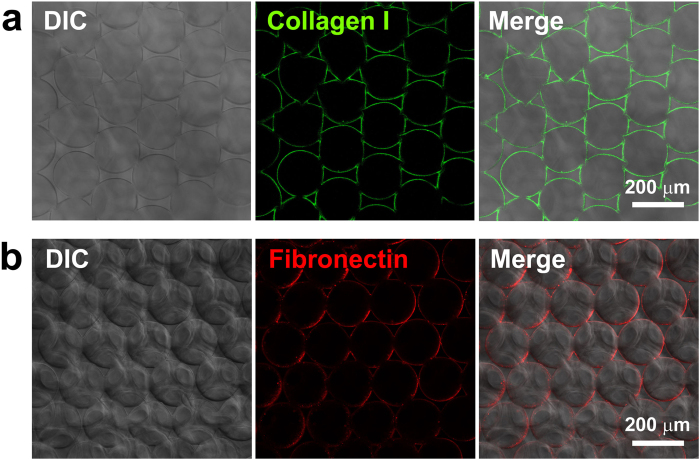
The coating of ECM proteins on the interior surface of scaffold cavities. (**a**) Collagen I was stained with green-fluorescence Alexa Fluor 488. (**b**) Fibronectin was stained with red-fluorescence Alexa Fluor 555. A uniform coating was achieved for both ECM proteins.

The fabrication and detailed characterization of collagen I functionalized-ICC scaffolds has been described previously^22^,^23^. As polystyrene beads with a 139 ± 2.9 μm diameter were used as the templates, a similar pore size was obtained in the PEG-DA ICC scaffolds. The pore size shrank to an average 102.3 ± 9.3 μm after freeze-drying, as measured on the scanning electron microscope (SEM) images^23^. Biofunctionalization with ECM protein, collagen I, or fibronectin to the interior surface of the PEG-NHS scaffold was accomplished by a coupling reaction between the NHS ester and amino groups on the protein^38^. As shown in Fig. 1, this method achieved a uniform coating of collagen I or fibronectin on the interior surface of scaffold cavities. For the purpose of direct comparison between collagen I and fibronectin, 20 μg/ml of each respective ECM protein was used to coat the ICC scaffolds and their effects on liver cell growth and specific liver functions were compared.

### Cell loading and proliferation in different ECM-functionalized scaffolds

**Figure 2:**
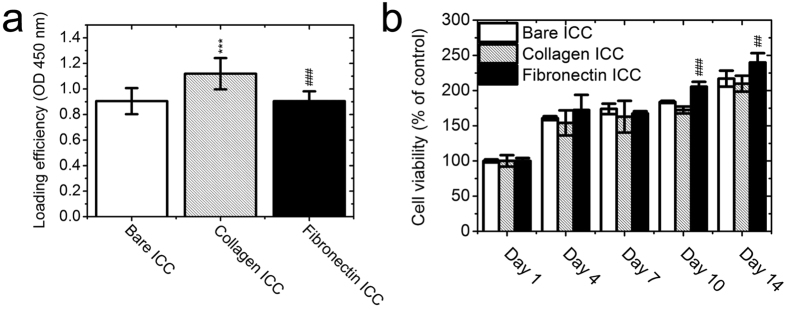
Loading efficiency and cell proliferation in ICC scaffolds. (**a**) 1 × 10^6^ cells were seeded into each piece of ICC scaffold and cell viability measured one day after cell seeding was used as the indicator of cell loading efficiency. (**b**) Cell viability was measured on days 1, 4, 7, 10, and 14 with the CCK-8 kit and proliferation was presented as the percentage of cell viability over day-1 value. The results are mean ± SD (N = 3). ***p < 0.001 compared with the bare ICC group; ^##^*p* < 0.01 compared with the collagen group; ^###^*p* < 0.001 compared with the collagen group.

**Figure 3:**
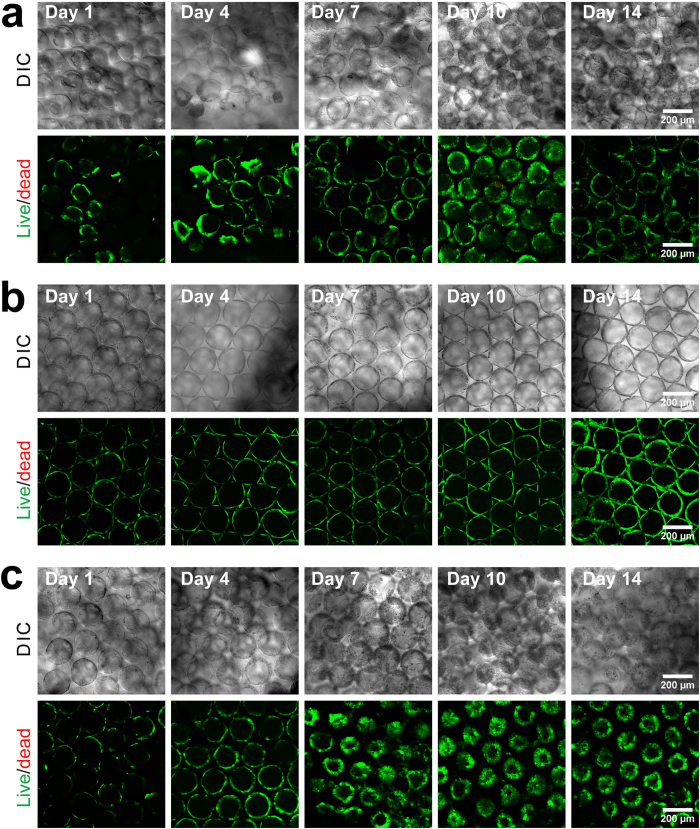
Live/dead staining images of cells in (**a**) the bare PEG-DA scaffold, (**b**) the collagen-functionalized scaffold and (**c**) the fibronectin-functionalized scaffold. Live cells were stained with green-fluorescence calcein and dead cells with red-fluorescence EthD-1. The results indicate that the majority of cells were alive until day 14 and that the cell number was increasing.

First, we investigated the effect of collagen I on cell loading efficiency in ICC scaffolds and directly compared it with fibronectin. Figure 2a shows that collagen I increased the cell loading efficiency of the ICC compared to the bare ICC scaffold (*p* < 0.001) but that the fibronectin coating did not. In fact, the cell loading efficiency of the fibronectin-functionalized ICC scaffold was relatively lower than the collagen I-functionalized scaffold (*p* < 0.001), indicating that Huh-7.5 cells did not adhere to the fibronectin as much as to the collagen I during initial cell seeding. Interestingly, although the initial cell attachment was poorer, cells on fibronectin-functionalized scaffolds proliferated slightly better than cells on collagen I-functionalized scaffolds, especially on days 10 and 14 (Fig. 2b). The live/dead staining images (Fig. 3) clearly illustrate that most cells were alive (green) up to day 14 in the three different groups tested, with only a small number of dead cells found on days 10 and 14 (scattered red dots). A marked difference was observed when we examined cell attachment patterns. The Huh-7.5 cell distribution was more random within the pores of the bare ICC scaffold whereas the cells were evenly attached to the surface of the collagen I-functionalized scaffold. This is consistent with previously reported results^22^,^23^. However, after the initial cell seeding, cell attachment to the fibronectin-functionalized ICC surface was not as evenly distributed as the attachment to the collagen I-functionalized surface. Later on (from day 4 until day 14), cells on the bare ICC scaffold grew toward the core of the cavities, with less growth observed along the cavity surfaces. Cells in the ECM-functionalized ICC scaffolds grew along the surface of cavities and the cell sheet thickened as the cells grew. It was evident that the cell sheets in the fibronectin group were significantly thicker than in those in the collagen I group, indicating a higher cell proliferation rate. This is consistent with the results presented in Fig. 2b and comparable to previously published results^22^,^23^.

### Collagen I and fibronectin differentially regulate liver-specific functions

**Figure 4:**
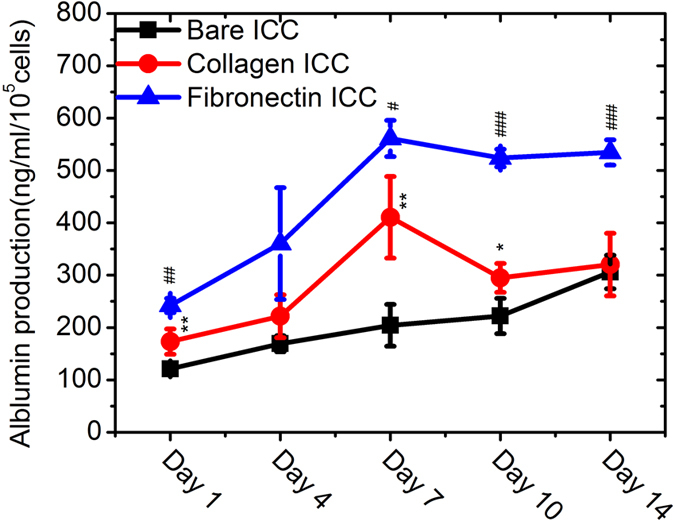
Albumin production by Huh-7.5 cells in the scaffolds. Data points are mean ± SD (N = 3). Comparison of the collagen-functionalized ICC and bare ICC groups: *p < 0.05; **p < 0.01. Comparison of the fibronectin-functionalized ICC and collagen-functionalized ICC groups: ^#^*p* < 0.05; ^##^*p* < 0.01; ^###^*p* < 0.001.

**Figure 5:**
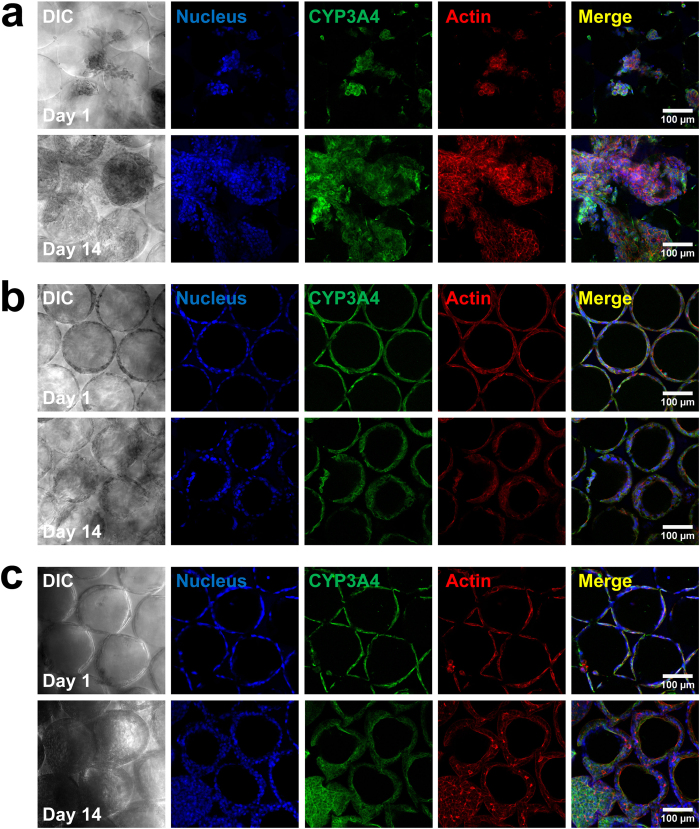
Immunofluorescence staining of CYP3A4 protein in Huh-7.5 cells on (**a**) the bare PEG-DA scaffold, (**b**) the collagen-functionalized scaffold, and (**c**) the fibronectin-functionalized scaffold, on days 1, 7, and 14. CYP3A4 was stained with Alexa Fluor 488 (green), F-actin was stained with Alexa Fluor 555 phalloidin (red), and cell nuclei were counterstained with DAPI (blue). High quality figures of the day 14 merged images are shown in Supplementary Fig. S1.

Next, we examined the influence of specific ECM proteins on the liver-specific functions of Huh-7.5 cells. Like primary hepatocytes, cultured Huh-7.5 cells synthesize and excrete albumin into the surrounding medium^39^. We collected the medium and measured accumulated albumin production by Huh-7.5 cells after 24 hours to characterize this important cellular function. As Fig. 4 shows, coating the ICC scaffold with either collagen I or fibronectin boosted albumin production compared to the bare ICC scaffold. The presence of ECM proteins provided an early advantage, as the difference in production between the ECM-coated groups and the bare ICC group was significant from day 1 onwards (*p* < 0.01). However, we did see a steady increase in albumin production in the bare ICC group until day 14, when production equaled that from the collagen I group. The fibronectin functionalized-ICC exhibited a much higher albumin production than either the collagen or the bare ICC groups, suggesting a distinct role of fibronectin on the regulation of Huh-7.5 cell functions. Another important functional marker of Huh-7.5 cells is the expression of intracellular metabolism enzymes, e.g., the cytochrome P450 family. We used immunofluorescence staining to probe for CYP3A4 protein expression in Huh-7.5 cells. Figure 5 shows the confocal microscope images of cells on the bare ICC (Fig. 5a), collagen I-functionalized ICC (Fig. 5b), and fibronectin-functionalized ICC (Fig. 5c), with CYP3A4 protein stained with green fluorescence. Unlike albumin production, CYP3A4 protein expression appears more similar among the different groups tested and was also consistent from day 1 to day 14, although this is not a very quantitative technique.

**Figure 6:**
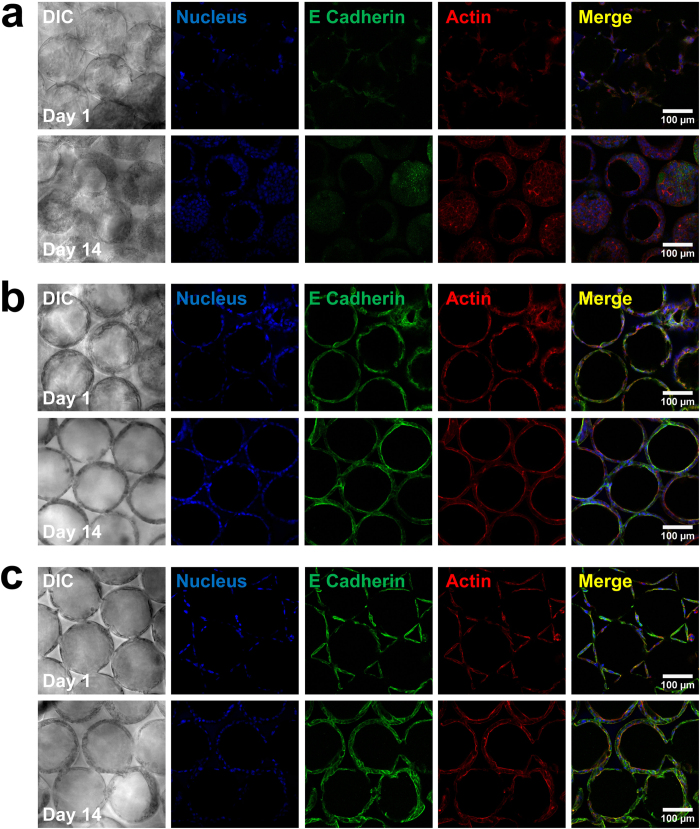
Immunofluorescence staining of E-cadherin in Huh-7.5 cells on (**a**) the bare PEG-DA scaffold, (**b**) the collagen-functionalized scaffold, and (**c**) the fibronectin-functionalized scaffold, on days 1, 7, and 14. E-cadherin was stained with Alexa Fluor 488 (green), F-actin was stained with Alexa Fluor 555 phalloidin (red), and cell nuclei were counterstained with DAPI (blue). High quality figures of the day 14 merged images are shown in Supplementary Fig. S2.

Due to the different cell attachment and growth patterns shown in Fig. 3, we decided to further investigate the underlying molecular mechanisms. E-cadherin is a calcium-dependent cell-cell adhesion protein that is highly expressed in epithelium^40^ as well as hepatocytes^41^. As it plays a key role in cellular adhesion, we used immunofluorescence staining to examine its expression level in the three types of scaffolds. Figure 6 shows markedly increased expression of E-cadherin in Huh-7.5 cells cultured in ECM protein-functionalized ICC scaffolds compared with the bare scaffold. The green fluorescence staining for E-cadherin in the bare ICC group was consistently dim from day 1 to day 14 (Fig. 6a), whereas there was a steady increase in green fluorescence intensity from day 1 to day 14 in both collagen I- and fibronectin-functionalized ICC groups (Fig. 6b,c). Importantly, the expression of E-cadherin was even higher in fibronectin-functionalized ICC than collagen I-functionalized ICC. The results suggest that E-cadherin could be involved in the formation of different morphologies of cell aggregation, regulated by collagen I or fibronectin.

### Collagen I and fibronectin differentially regulate the gene expression patterns of Huh-7.5 cells

**Figure 7:**
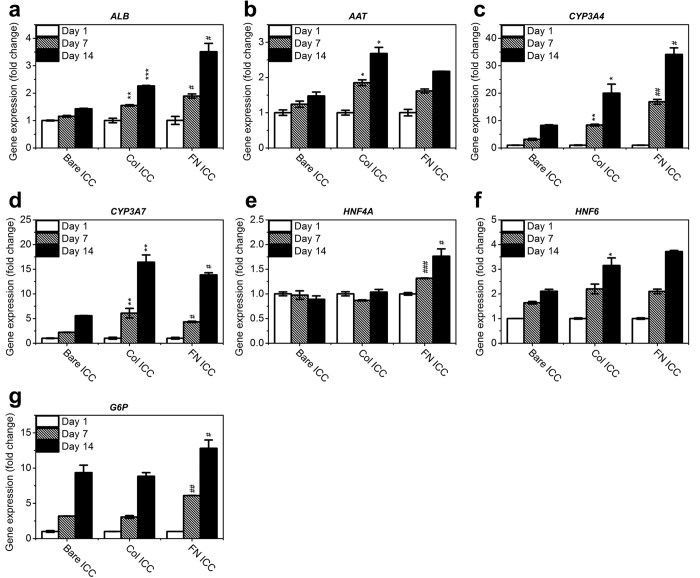
Gene expression of liver functional markers in Huh-7.5 cells on ICC scaffolds. Quantitative real-time PCR analysis of the mRNA of specific genes was conducted, and the data were normalized to the housekeeping gene GAPDH. The results are presented as the fold change in the day 1 value of each respective group (mean ± SD, N = 3). Fold change comparison between the collagen-functionalized ICC and bare ICC groups: *p < 0.05; **p < 0.01; ***p < 0.001. Comparison of the fibronectin-functionalized ICC and collagen-functionalized ICC groups: ^#^*p* < 0.05; ^##^*p* < 0.01; ^###^*p* < 0.001.

**Figure 8:**
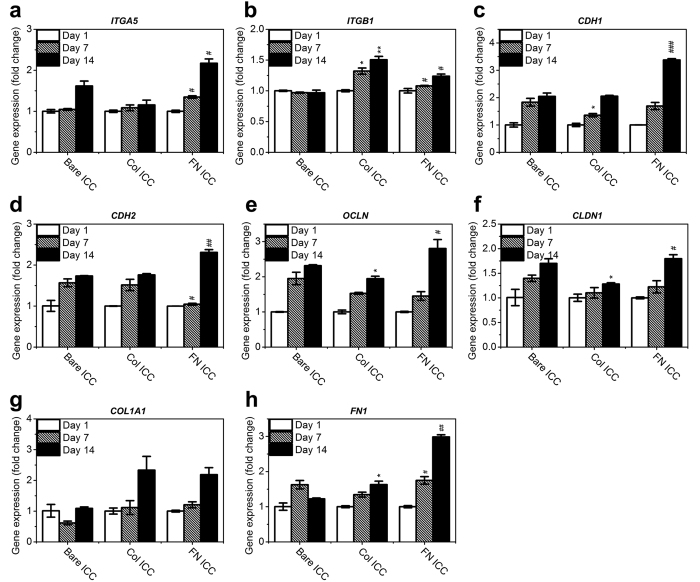
Gene expression of cell-cell and cell-ECM signaling-related proteins in Huh-7.5 cells on ICC scaffolds, with or without collagen or fibronectin functionalization. The data were normalized to the housekeeping gene GAPDH. The results are presented as the fold change of the day 1 value of each respective group (mean ± SD, N = 3). Fold change comparison between the collagen-functionalized ICC and bare ICC groups: *p < 0.05; **p < 0.01; ***p < 0.001. Comparison between the fibronectin-functionalized ICC and collagen-functionalized ICC groups: ^#^*p* < 0.05; ^##^*p* < 0.01; ^###^*p* < 0.001.

To better understand the effects of specific ECM proteins on the cell phenotype, we used quantitative PCR to analyze the expression levels of a wide array of genes related to liver-specific functions (Fig. 7) or cell-cell/cell-ECM signaling (Fig. 8). The liver-specific functional genes include *ALB* (encoding albumin), *AAT* (alpha 1-antitrypsin), *CYP3A4* (cytochrome P450 3A4), *CYP3A7* (cytochrome P450 3A7), *HNF4A* (hepatic nuclear factor 4-alpha), *HNF6* (hepatic nuclear factor 6), and *G6P* (glucose 6-phosphatase). As stated earlier, albumin production is one of the most important functions of liver cells^42^. The expression and metabolic activity of cytochrome P450 proteins, including CYP3A4 and CYP3A7, are also important markers of hepatocyte function^43^. Alpha 1-antitrypsin is a protease inhibitor that is produced by the liver and released into the serum^44^ and glucose 6-phosphatase, also primarily produced by the liver, is an enzyme that hydrolyzes glucose-6-phosphate^45^. Hepatocyte nuclear factors, including HNF4α and HNF6, are a group of transcription factors that are predominately expressed in the liver^46^. Figure 7 shows a steady increase in gene expression levels from day 1 to day 14 across all scaffolds, for most of the liver functional genes except for *HNF4A* (Fig. 7E), which increased with time only in the presence of fibronectin. Both collagen I and fibronectin facilitated the gene expression of *ALB*, *AAT*, *CYP3A4*, *CYP3A7*, and *HNF6* compared to the bare scaffold. The gene expression levels of *ALB*, *CYP3A4*, *HNF4A*, and *G6P* in the fibronectin group were significantly higher than in the collagen I group (*p* < 0.05) whereas the expression of *CYP3A7* was significantly lower (*p* < 0.05). For *CYP3A4*, we noticed a discrepancy between the mRNA expression level determined by RT-qPCR and the protein expression level reflected by the qualitative immunofluorescence data (Fig. 5). In fact, it is a common observation that protein abundance and mRNA expression levels in biological samples do not always correlate well for various reasons, such as the complicated post-transcriptional mechanisms involved in protein synthesis, different half-lives for mRNA and protein, and error and noise in both protein and mRNA experiments^47^.

The cell signaling-related genes we analyzed were *ITGA5* (integrin alpha-5), *ITGB1* (integrin beta-1), *CDH1* (E-cadherin), *CDH2* (N-cadherin), *OCLN* (occludin), *CLDN1* (claudin-1), *COL1A1* (collagen type I alpha 1), and *FN1* (fibronectin 1). Integrins are transmembrane receptors involved in both cell-cell and cell-ECM interactions^48^; cadherins are the most important cell-cell adhesion proteins^49^; both occludin and claudin-1 are intercellular tight junction proteins, as well as the co-receptors for hepatitis C virus infection^50^,^51^; and collagen and fibronectin are the most important ECM proteins^27^. Figure 8 shows that the presence of collagen I resulted in a higher expression level of *ITGB1* (*p* < 0.05) and lower expression levels of *OCLN* and *CLDN1* (*p* < 0.05 on day 14) compared to the bare ICC scaffold. Interestingly, we found a more significant difference between the fibronectin and collagen I group: in the fibronectin group, the expression levels of *ITGA5*, *CDH1*, *CDH2*, *OCLN*, *CLDN1* and *FN1* were higher, but the level of *ITGB1* was consistently lower.

## Discussion

The interaction between cells and ECM regulates important cellular behavior and functions. The differential effect of specific ECM components (e.g. collagens, fibronectin, and laminin) on hepatocyte behavior has been studied in monolayer cell culture^33^,^34^,^35^,^36^,^37^, but is not well understood in 3D culture. In our previous study, we compared the morphology and functions of liver-derived cells in 2D monolayer culture and 3D ICC scaffolds, and further examined the difference depending on the modifications of the ICC scaffold cavity surface: namely, bare PEG-DA hydrogel, NHS functionalization, or different densities of collagen I functionalization^22^. As expected, a collagen I-functionalized scaffold represented the most nurturing environment for the growth of Huh-7.5 cells and the induction of liver-specific functions because this protein is an essential element of the liver ECM. Although cells have the capability to remodel their surrounding environment by synthesizing and excreting ECM proteins, such processes may depend on initial stimulation from existing ECM. This phenomenon is well documented in the literature^52^; it was also confirmed by our previous study^22^, in which we found that Huh-7.5 cells were able to produce much more collagen and fibronectin when cultured in a collagen-functionalized ICC scaffold. Similarly, in the current study we observed that both collagen and fibronectin mRNAs were highly expressed in Huh-7.5 cells growing on the ECM-functionalized ICC scaffolds (Fig. 8g,h), representing an interesting positive feedback loop.

The current study is focused on elucidating the differences between fibronectin and collagen in the regulation of cell phenotype in a 3D scaffold. Fibronectin mediates the binding with integrins on the cell membrane; it is also recognized that fibronectin binds to collagen on the ECM basal membrane^53^. In other words, fibronectin may form a bridge between cells and collagen. This could provide an explanation for the reported enhanced spreading of hepatocytes on a fibronectin surface compared to a collagen surface^33^. In our case, compared to collagen, fibronectin directed different attachment and growth patterns of Huh-7.5 cells in the 3D scaffold (Fig. 3), although it was difficult to discern improved “spreading” based on the z-section images taken with a confocal microscope. More striking was that the albumin production rate by cells on a fibronectin scaffold was almost 80% higher than production from cells on either a collagen scaffold or a bare scaffold (Fig. 4, days 10 and 14), in parallel with the *ALB* gene expression levels (Fig. 7a). This observation confirmed previous findings from a study of rat hepatocytes in a monolayer culture^36^. These data were also consistent with the up-regulation of HNF-4α, which is required for liver development and the expression of many liver-specific genes including cytochrome P450, in the fibronectin scaffold (Fig. 7e).

Cellular functions are invariably connected to the position of the cell in the microenvironment. Depending on the nature of the substrate, whether neutral PEG hydrogel or adhesion-promoting collagen/fibronectin, cells respond to such an environment by (i) expressing integrins to mediate cell-ECM adhesion, (ii) producing their own ECM proteins, and (iii) expressing cadherins, occludin, and claudins to mediate cell-cell adhesion and intercellular tight junctions. It was therefore not surprising to discover that many of these cell adhesion-related genes were differentially expressed in different types of scaffolds (Fig. 8). As a result, cells in these scaffolds displayed different aggregation morphologies (Fig. 3). Furthermore, these different environmental cues likely contributed to the functional differences between the fibronectin and collagen groups.

Both ECM proteins and 3D geometry are key environmental factors for the proper culture of cells. In this study, we examined in detail the differences between two ECM component proteins, fibronectin and collagen, in the phenotypic regulation of Huh-7.5 human hepatocyte-derived cells cultured in scaffolds with highly ordered cavities. The functionalization of the microporous ICC scaffold with either fibronectin or collagen resulted in different cell morphologies, functions, and gene expression patterns. Specifically, fibronectin was superior to collagen in promoting albumin production and the expression of certain liver-specific genes, including *ALB*, *CYP3A4*, *HNF4A*, and *G6P*. A marked difference was also observed in the expression of cell adhesion-related genes, highlighting a distinct role of fibronectin, in contrast to collagen, in liver cell organization and function. These results of cell-ECM interactions have great implication for future studies involving primary hepatocytes, and could provide essential insights to the establishment of *in vitro* liver models.

## Methods

### Fabrication of PEG-DA-based bare ICC scaffold

Poly(ethylene glycol) diacrylate (PEG-DA) was synthesized from diol-terminated PEG (MW = 4.6 kDa; Sigma-Aldrich, MO) following a previously published protocol^54^. The fabrication of PEG-DA-based inverted colloidal crystal (ICC) has been published elsewhere^22^,^23^. Briefly, Eppendorf tubes (internal diameter 6 mm; Axygen Scientific, NY) glued to a glass slide were used as molds for colloidal crystal formation. Polystyrene (PS) beads (139 ± 2.9 μm; Duke Scientific, CA) suspended in 70% ethanol were pipetted into the molds and repeatedly washed with ethanol. The PS beads in the molds were sonicated for 3 minutes to become orderly stacked colloidal crystals (CCs). After the ethanol had completely evaporated, the CC of PS beads were sintered in a furnace by heating at 130 °C for 6 h and then removed from the molds. A solution of 50% (*w/v*) PEG-DA and 0.05% (*w/v*) 2-hydroxy-4′-(2-hydroxyethoxy)-2-methylpropiophenone (photo initiator; Sigma-Aldrich, MO) in deionized water was infiltrated into the gaps of CCs via centrifugation. The composite of CC and pre-polymer solution was cured by 5 min irradiation under a UV lamp (365 nm, 10.84 mW/cm^2^). PS spheres were then removed by tetrahydrofuran (THF) dissolution and subsequently washed with 70% ethanol and phosphate-buffered saline (PBS; pH 7.4).

### Biofunctionalization of ICC scaffolds with collagen I or fibronectin

A similar protocol to that described above was used to produce an N-hydroxysuccinimide (NHS) ester-functionalized ICC scaffold, with a pre-polymer solution containing 50% (*w*/*v*) PEG-DA, 10% (*w*/*v*) acryloyl-PEG-NHS (Laysan Bio, AL), and 0.05% photo initiator in deionized water. The prepared PEG-NHS ICC scaffold was coated with either collagen I or fibronectin by centrifugation, shaking, and incubation in 20 μg/ml collagen I (Merck Millipore, Germany) or fibronectin (Merck Millipore) solution in pH 7.4 PBS at 4 °C overnight. Excess collagen I or fibronectin was removed by repeated washing with PBS.

To characterize the ECM coating, the ICC scaffolds were fixed in 4% paraformaldehyde (Alfa Aesar, MA) then incubated with a mouse primary antibody against collagen I or fibronectin (Abcam, UK) at 4 °C overnight in the presence of 3% bovine serum albumin (BSA; Sigma-Aldrich) and labeled with an anti-mouse secondary antibody conjugated to a fluorophore. Green fluorescent Alexa Fluor 488 was used to label collagen I and red fluorescent Alexa Fluor 555 was used to label fibronectin. After PBS washing, the ECM-functionalized scaffolds were imaged using a Carl Zeiss LSM 710 confocal microscope.

### Huh-7.5 cell culture and seeding in the ICC scaffolds

Huh-7.5 hepatocellular carcinoma cells (Apath, NY) were cultured in Dulbecco’s Modified Eagle’s Medium (DMEM; Hyclone, UT) supplemented with 10% fetal bovine serum (FBS; Hyclone), 100 U/ml penicillin and 100 μg/ml streptomycin (Life Technologies, MA). Cultures were maintained at 37 °C in the presence of 5% CO_2_. To ensure efficient cell seeding, ICC scaffolds were preconditioned in complete growth medium for 30 min then slightly dried and sterilized with 1-h UV irradiation. Confluent Huh-7.5 cells were trypsinized with 0.25% trypsin-EDTA (Gibco), collected, counted, and then resuspended in complete growth medium at a concentration of 4 × 10^7^ cells/ml. Cell seeding was accomplished by pipetting 25 μl cell suspension (containing 1 × 10^6^ cells) onto each piece of ICC scaffold and incubating for 12 hours. Subsequently, the cell-laden scaffolds were transferred to a new 24-well plate containing 2 ml medium in each well, and maintained at 37 °C/5% CO_2_ with fresh medium changed every 3 days.

### Cell viability, loading efficiency, and proliferation

Cell viability was quantified with a colorimetric assay using the Cell Counting Kit-8 (CCK-8; Dojindo Molecular Technologies, MD). For the comparison of cell loading efficiency in different scaffolds, cell-laden scaffolds were transferred to a new 24-well plate one day after cell seeding. The scaffolds were incubated in complete medium containing CCK-8 reagent for 2 h at 37 °C then the supernatants were transferred to a 96-well plate. The absorbance at 450 nm was measured on an Infinite 200 PRO microplate reader (Tecan, Switzerland).

Cell proliferation in the ICC scaffold was assessed by measuring cell viability with the CCK-8 assay on days 1, 4, 7, 10, and 14 after seeding then normalizing the absorbances to the day 1 value to calculate the percentage of cell growth. The absorbance value was used to represent cell number based on a presumably linear relationship between cell number and metabolic activity.

Cell viability was also visually assessed with the LIVE/DEAD viability/cytotoxicity kit (Life Technologies, CA) following the manufacturer’s manual. Calcein-AM and ethidium homodimer-1 (EthD-1) were added to complete medium at 4 μM and 8 μM respectively; this supplemented medium was then incubated with the cell-laden ICC scaffolds for 1 h at 37 °C. Live cells were stained with green fluorescence (calcein) and dead cells were stained red (EthD-1). Fluorescence images were taken using a confocal microscope.

### Albumin secretion

Albumin secretion by Huh-7.5 cells was analyzed with a human albumin ELISA kit (Abcam), following the manufacturer’s protocol. To collect the secreted albumin, the cell culture medium was changed in each well 24 h before collection, then the medium was collected on days 1, 4, 7, 10, and 14. Collected media were stored at −80 °C until the albumin measurement was carried out. The results are presented as the normalized fold change compared with the day 1 value of the same group.

### Immunofluorescence staining

Cellular CYP3A4 and E-cadherin protein expressions were visually analyzed with immunofluorescence staining and subsequent confocal microscopy. Briefly, on days 1, 7, or 14, cell-laden ICC scaffolds were removed from 24-well plates, washed with PBS, then fixed in 4% paraformaldehyde (PFA) for 5 min. Next, cells were permeabilized with a solution of 0.1% Triton X-100 (Bio-Rad, CA) in PBS for 30 min, washed with PBS, and incubated in 3% BSA blocking buffer for 1 h. Cells were then incubated with mouse monoclonal primary antibodies against CYP3A4 or E-cadherin (Santa Cruz Biotechnology, CA) overnight at 4 °C, washed with PBS, and incubated with anti-mouse secondary antibody conjugated to Alexa Fluor 488 (Life Technologies) for 2 h at room temperature. F-actin was stained with Alexa Fluor 555 phalloidin (Life Technologies). Lastly, cell nuclei were counterstained with 10 μg/ml 4′,6-diamidino-2-phenylindole (DAPI; Life Technologies) for 5 min. Fluorescence images were immediately taken using a confocal microscope.

### Realtime PCR

Total RNA was extracted from Huh-7.5 cells with TRIzol reagent (Life Technologies) following the manufacturer’s instructions. RNA concentration was measured with a NanoDrop spectrophotometer (Thermo Scientific, MA) and cDNA was synthesized with iScript Reverse Transcription Supermix (Bio-Rad). Real-time quantitative polymerase chain reaction (qPCR) was performed with the CYBR Select Master Mix for CFX (Thermo Fisher Scientific) on a CFX Connect Real-Time PCR Detection System (Bio-Rad). The following amplification mode was used for qPCR: the first 20 s at 95 °C then 40 cycles of 10 s at 95 °C and 40 s at 60 °C. We analyzed the expression of the following liver function genes on days 1, 7, and 14: (1) albumin (*ALB*), (2) alpha 1-antitrypsin (*AAT*), (3) cytochrome P450 3A4 (*CYP3A4*), (4) cytochrome P450 3A7 (*CYP3A7*), (5) hepatocyte nuclear factor 4-alpha (*HNF4A*), (6) hepatocyte nuclear factor 6 (*HNF6*), and (7) glucose 6-phosphatase (*G6P*). We also analyzed the expression of the following cell adhesion/signaling related genes: (1) integrin alpha-5 (*ITGA5*), (2) integrin beta-1 (*ITGB1*), (3) E-cadherin (*CDH1*), (4) N-cadherin (*CDH2*), (5) occludin (*OCLN*), (6) claudin-1 (*CLDN1*), (7) collagen (*COL1A1*), and (8) fibronectin (*FN1*). The gene expression results were normalized to the *GAPDH* expression level, and presented as the normalized fold change relative to the day 1 value of the appropriate group. The relative expression was calculated by the 2^−ΔΔCt^ method. The sequences of the primers used for cDNA synthesis are listed in Table 1.

### Statistical analysis

All the data are presented as mean ± SD. The differences between multiple groups were determined by one-way ANOVA. A *p-*value < 0.05 was considered statistically significant.

## Additional Information

**How to cite this article**: Wang, Y. *et al*. ECM proteins in a microporous scaffold influence hepatocyte morphology, function, and gene expression. *Sci. Rep.* **6**, 37427; doi: 10.1038/srep37427 (2016).

**Publisher’s note:** Springer Nature remains neutral with regard to jurisdictional claims in published maps and institutional affiliations.

## Supplementary Material

Supplementary Information

## Figures and Tables

**Table 1:** Primer sequences used in RT-qPCR.

Target gene	Forward (5′-3′)	Reverse (5′-3′)
*Liver functional marker*		
*ALB*	CTGCACAGAATCCTTGGTGA	CTCCTTATCGTCAGCCTTGC
*AAT*	GATGCTGCCCAGAAGACAGA	GGAGTTCCTGGAAGCCTTCA
*G6P*	TTCCTGTTCAGCTTCGCCAT	TCAAAGACGTGCAGGAGGAC
*CYP3A4*	ACCGTGACCCAAAGTACTGG	TTCAGGGGGATCTGTGTTTC
*CYP3A7*	AAGTGGACCCAGAAACTGCA	GGCTCCACTTACGGTCTCAT
*HNF4A*	TCGTTGAGTGGGCCAAGTAC	TGTCATCGATCTGCAGCTCC
*HNF6*	TTGAGCCATTGAGCGGACAT	GGCAGGTTCAAACGTTAGGC
*Cell signaling-related gene*		
*ITGA5*	CAGATCCTGTCTGCCACTCA	GAGGGATCGAATGTCTGAGC
*ITGB1*	GAAGGGCGTGTTGGTAGACA	GTTGCACTCACACACACGAC
*CDH1*	AGGCCAAGCAGCAGTACATT	AAATGTGTCTGGCTCCTGGG
*CDH2*	CCTTTCAAACACAGCCACGG	TGTTTGGGTCGGTCTGGATG
*OCLN*	GAGTTGACAGTCCCATGGCA	CCGCCAGTTGTGTAGTCTGT
*CLDN1*	TGGAAGACGATGAGGTGCAG	GCTGGAAGGTGCAGGTTTTG
*COL1A1*	TTCTGCAACATGGAGACTG	ATGTAGGCCACGCTGTTCT
*FN1*	GGCAACGTGTTACGATGAT	CGGGAATCTTCTCTGTCAG
*GAPDH*	CCATGGGGAAGGTGAAGGTC	CTCGCTCCTGGAAGATGGTG
