# Supplementary material for: ECM proteins in a microporous scaffold influence hepatocyte morphology, function, and gene expression
Source: Sci Rep. 2016 Nov 29;6:37427. doi: 10.1038/srep37427 (PMC5126637; doi:10.1038/srep37427)

Supplementary Information

ECM proteins in a microporous scaffold influence hepatocyte morphology, function, and gene expression

Yan Wang[1,2,±], Myung Hee Kim[1,2,±], Hitomi Shirahama[1,2], Jae Ho Lee[1,2], Soon Seng Ng[1,2], Jeffrey S. Glenn[3,4] & Nam-Joon Cho[1,2,5,*]

[1]School of Materials Science and Engineering, Nanyang Technological University, 50 Nanyang Avenue 639798, Singapore

[2]Centre for Biomimetic Sensor Science, Nanyang Technological University, 50 Nanyang Drive 637553, Singapore

[3]Division of Gastroenterology and Hepatology, Department of Medicine, Stanford University School of Medicine, Palo Alto, California, United States of America

[4]Department of Microbiology and Immunology, Stanford University School of Medicine, Palo Alto, California, United States of America

[5]School of Chemical and Biomedical Engineering, Nanyang Technological University, 62 Nanyang Avenue 637459, Singapore

[±]These authors contributed equally to this work.

[*]Correspondence and requests for materials should be addressed to N.J.C. (email: njcho@ntu.edu.sg)

Supplementary Figure 1. High quality figures of the day 14 merged images of Huh-7.5 cells with immunofluorescence staining for CYP3A4 (green) and co-stained for F-actin (red) and nuclei (blue).

Supplementary Figure 2. High quality figures of the day 14 merged images of Huh-7.5 cells with immunofluorescence staining for E-cadherin (green) and co-stained for F-actin (red) and nuclei (blue).

Supplementary Figure 1

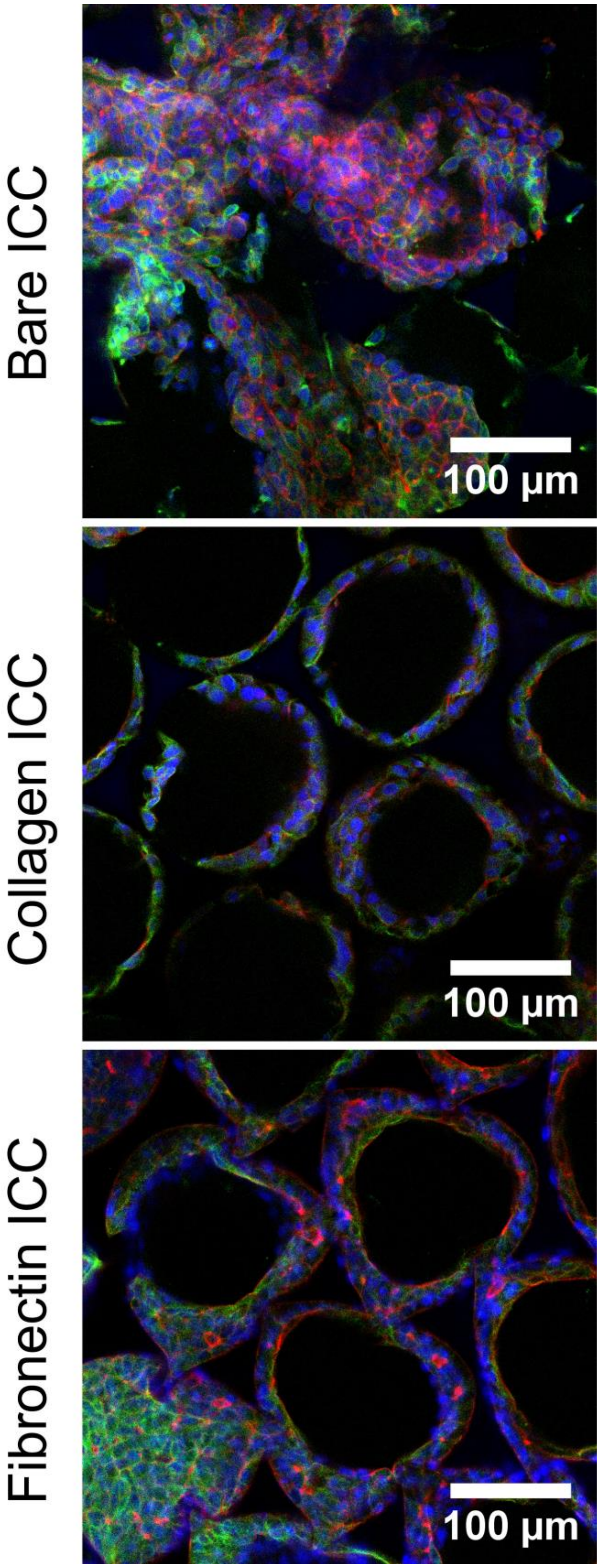

Supplementary Figure 2

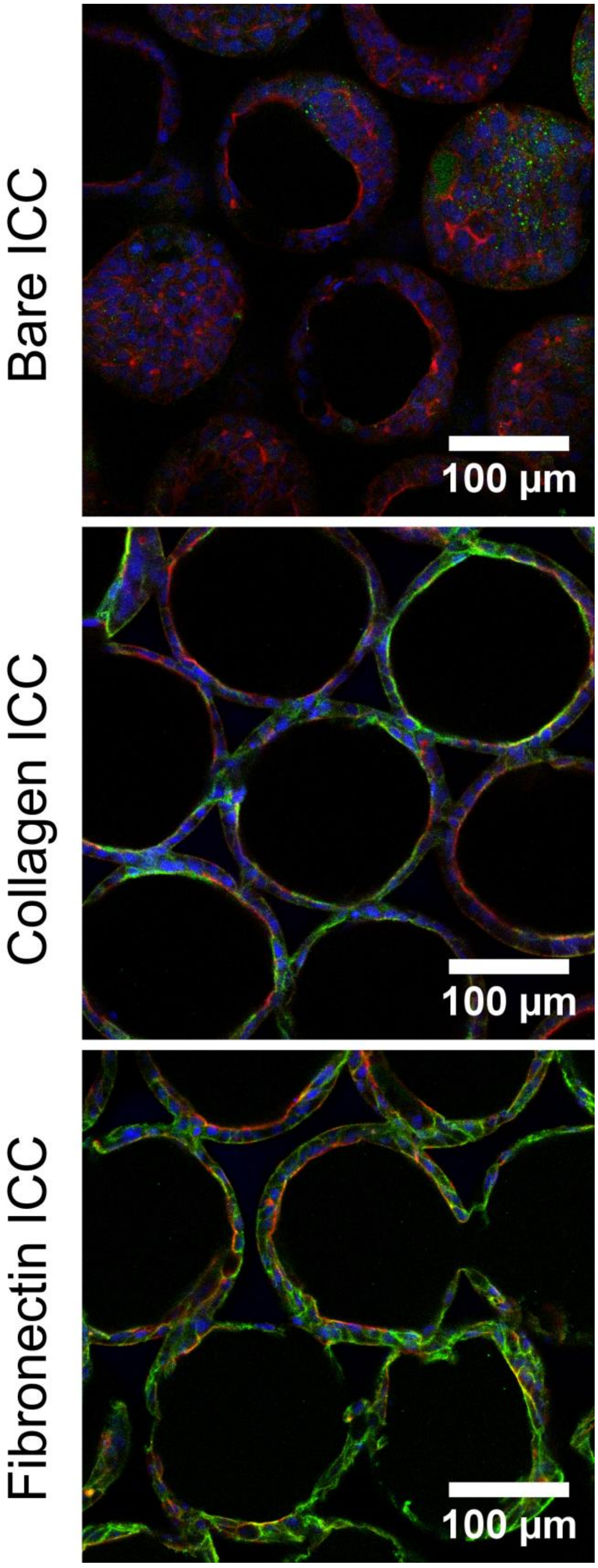

Supplement: Supplementary Information [file srep37427-s1.pdf]
